# Anti-signal recognition particle positive necrotizing myopathy-sjogren’s syndrome overlap syndrome: a descriptive study on clinical and myopathology features

**DOI:** 10.1186/s12891-023-06354-5

**Published:** 2023-03-23

**Authors:** Li Xu, Meng-ge Yang, Liya Hu, Huajie Gao, Suqiong Ji

**Affiliations:** 1 Department of Neurology, Tongji Hospital of Tongji Medical College, Huazhong University of Science and Technology, Jiefang Street 1095#, Wuhan, 430000 China; 2 Department of Geriatrics, Tongji Hospital, Tongji Medical College, Huazhong University of Science and Technology, Wuhan, Hubei China

**Keywords:** Immune-mediated necrotizing myopathy, Anti-signal recognition particle antibodies, Sjogren’s syndrome, Overlap syndrome

## Abstract

**Background and objective:**

The aim of this study was to elucidate the clinical and myopathological characteristics of patients with anti-signal recognition particle (SRP) positive immune-mediated necrotizing myopathy (IMNM) overlap Sjogren’s syndrome (SS).

**Materials and methods:**

We retrospectively analyzed the data of anti-SRP positive IMNM patients admitted in the Neurology Department of Tongji Hospital between January 2011 to December 2020. Patients were divided into two groups: anti-SRP IMNM overlap SS group and anti-SRP IMNM control group. The clinical features, laboratory results, histological features, treatment, and prognosis were compared between the two groups.

**Results:**

A total of 30 patients with anti-SRP IMNM were included, including six anti-SRP IMNM overlap SS patients (two males, four females), with a median age of 39 years, and 24 anti-SRP IMNM patients (ten males, fourteen females), with a median age of 46 years. The anti-SRP IMNM overlap SS group had a lower prevalence of muscle atrophy (0 vs 50%, *p* = 0.019), and a higher prevalence of extramuscular manifestations, including cardiac abnormalities and ILD (Interstitial lung disease). CD4 + and CD68 + inflammatory infiltrations were significantly increased in anti-SRP IMNM overlap SS patients, with an increased presence of CD4 + cells in both necrotic(*p* = 0.023) and endomysial areas (*p* = 0.013), and more CD68 + cells (*p* = 0.016) infiltrated the endomysial area. Deposition of membrane attack complex (MAC) on sarcolemma (*p* = 0.013) was more commonly seen in the anti-SRP IMNM overlap SS group.

**Conclusion:**

Our data revealed that anti-SRP IMNM-SS overlap patients may present with milder muscular manifestation, but worse extramuscular manifestations compared to anti-SRP IMNM patients without SS. CD4 + and CD68 + inflammatory infiltrations and MAC deposition were remarkably increased in anti-SRP IMNM-SS overlap patients.

**Supplementary Information:**

The online version contains supplementary material available at 10.1186/s12891-023-06354-5.

## Key Message

Anti-SRP IMNM-SS overlap patients may present with milder muscular manifestation but worse extramuscular manifestations. CD4 + and CD68 + inflammatory infiltrations and MAC deposition were more prevalent in anti-SRP IMNM-SS overlap patients. Germinal center-like structures of the lymphocytic foci histologic characteristics might be a specific characteristic for overlap SS patients.

## Introduction

Immune-mediated necrotizing myopathy (IMNM) is a known subgroup of idiopathic inflammatory myopathies (IIMs), characterized by proximal weakness, elevated creatine kinase, and necrosis with or without lymphocytic infiltrates on muscle specimens [1]. The 2016 European Neuromuscular Centre (ENMC) -IMNM categorizes IMNM into three subgroups according to positive antibodies: anti-SRP IMNM, anti-HMGCR IMNM, and seronegative IMNM, the latter of which has no myositis-specific autoantibodies and must be diagnosed with a muscle biopsy. Among the IIMs, the incidence of anti-SRP IMNM ranged from 5 to 15% [1]. Anti-SRP-associated IMNM is usually more severe [2], with more intense muscle damage [3] and has a worse outcome than other subtypes of IMNM.

Sjögren’s syndrome (SS) is an autoimmune disease predominantly characterized by lymphocytic and plasmocytic infiltrations of the exocrine glands, leading to dryness of the eyes and mouths [4]. The prevalence of myopathy in SS patients has been reported to vary from 1 to 10%. The study of myositis-SS overlap had focused on polymyositis (PM), dermatomyositis (DM) and inclusion body myositis (IBM) [5]. Although anti-SRP IMNM overlap SS had been reported in a few cases [6], the prevalence of concurrent presentation of these conditions is unknown, and muscle biopsy data to confirm muscle histopathology characteristics and detailed distribution of anti-SRP IMNM overlap SS is rare.

The aim of this study was to summarize the clinical and myopathological characteristics of anti-SRP IMNM overlap SS, by comparing such patients with anti-SRP IMNM controls. A better understanding of characteristics of anti-SRP IMNM overlap SS maybe helpful to ensure early diagnosis and treatment of inflammation, to prevent disease progression, and improve long-term outcomes.

## Materials and methods

### Patient selection

Anti-SRP-associated IMNM patients were enrolled in the Department of Neurology at Tongji Hospital from January 2011 to December 2020. The diagnostic criteria of anti-SRP-associated IMNM were based on ENMC International Workshop on Idiopathic Inflammatory Myopathies [7]. Anti-SRP-associated IMNM patients with SS fulfilled the 2016 American College of Rheumatology/European League Against Rheumatism classification criteria [8]. The disease duration of anti-SRP IMNM overlap SS patients was defined as the period between symptoms of muscle weakness initial shown up and anti-SRP IMNM overlap SS patients diagnosis. The exclusion criteria were as follows: (1) autoimmune serologic tested negative for anti-SRP; (2) insufficient clinical data. According to the above criteria, anti- SRP-associated IMNM patients were divided into two groups: the anti-SRP IMNM overlap SS group and the anti-SRP IMNM control group.

This study was approved by the Ethics Committee of Tongji Hospital (IRB ID: TJ-C20121221), all patients provided written informed consent.

### Clinical data and treatment outcome measures

Clinical data were collected from Electronic Medical Record System, including demographic information, clinical characteristics (disease duration, muscle strength, atrophy, rash, dysphagia, dyspnea, myalgia), and laboratory results. Patients were classified according to the MRC (Medical Research Council) [9] grade of the weakest muscle group, as follows: none (MRC grade 5), mild (MRC grade ≥ 4/5), moderate (MRC grade 3–4/5), and severe (MRC grade < 3/5). Manual Muscle Testing (MMT) score of 26 muscle groups, Disease Activity Score (DAS) [10], Myositis disease activity assessment visual analogue scale (MYOACT) [11] were also performed. All the tests were performed by the same neurologist. Prognosis was graded as no improvement, improvement (> 1 MRC grade in multiple muscle groups, demanding minimal assistance with activities of daily living), marked improvement (symptoms and signs of mild weakness, and normal or near normal functioning) [12].

We further evaluated the following clinical indices in all patients once every three months: serum CK levels, LDH, myoglobulin, renal and hepatic function, and blood glucose, regular laboratory results (routine blood and urine tests). Relapse was determined when the symptoms reappeared or CK levels significantly increased after the patient achieved remission.

### Autoimmune serologic testing

Serum samples from all the patients were tested for rheumatoid-related factors, myositis special antibodies (MSAs), and myositis associated antibodies (MAAs). The rheumatoid-related factors, including anti-nuclear, anti-SSA/Ro60, anti-SSB/La, anti-Sm, anti-RNP, anti-mitochondrial, anti-dsDNA antibodies, were performed at Tongji Hospital Laboratory, The following MSAs and MAAs were estimated using two commercial semi-quantitative line blot assays (D-Tek, Germany; Euroline, Germany) with the following antibodies: anti-SRP, anti-HMGCR, anti-Ro52, anti-Mi2α and β, anti-TIF1γ, anti-MDA5, anti-NXP2, anti-SAE1, anti-Jo1, anti-PL7, anti-PL12, anti-Ku, anti-EJ, anti-OJ, anti-cN-1A, anti-PMScl100 and anti-PMScl75 antibodies [13]

### Biopsy specimens and immunohistochemistry

Skeletal muscle biopsies were performed in all patients. Consecutive cryostat sections of muscle samples (7 μm thick) were stained using routine methods, including haematoxylin and eosin, modified Gomori’s trichrome, myosin ATPase, NADH-tetrazolium reductase, succinate dehydrogenase, cytochrome C oxidase, periodic acid-Schiff, acid phosphatase, oil red O enzymatic, Sudan black. The immunological characteristics and inflammatory infiltrates were performed by immunohistochemical staining using anti-CD20 (1:50, A4893, Abclonal), anti-CD4 (1:100, A19018, Abclonal) and anti-CD8 (1:4000, 66,968–1-Ig, Proteintech), anti-CD68 (1:100, sc-20060, Santa), anti-Major Histocompatibility Complex class I (MHC class I) (1:400, ab22432, Abcam), anti-C5b-9 (Membrane attack complex; MAC) antibody (1:200, sc-58935, Santa), anti-CD23 (1:100, 60,208–1-Ig, Proteintech), Activation-induced cytidine deaminase (AID) (1:200, YT5566, North American ImmunoWay Biotechnology Company). All sections were observed using a microscope (BX53, Olympus) under light conditions and photographed by Hamamatsu NanoZoomer S360.

### Quantification of immunohistochemical staining

For quantification of CD4 + , CD8 + , CD20 + lymphocytes, and CD68 + macrophages, sections stained for MAC, MHC class I ((MAC and MHC class I grade range: 0–3, additional file 1) were analyzed and graded in a semi-quantitative manner which was adopted with some modifications. Analysis was performed by two myopathologists using traditional microscopy evaluation, manual cell counts of 10 randomly selected high-power fields (HPFs, magnification × 400) were used to calculate the average number of cells per section for quantification. Whole slide images were obtained with a virtual microscope using ImageJ.

### Statistics assessments

All date analysis were performed using SPSS version 23.0, and figures were plotted using GraphPad PRISM software version 9.0 (Graph Pad Software Inc., San Diego, CA, USA, 2020). T-test were conducted for continuous variable and Chi-square tests were conducted for categorical variables to compare differences between the two groups. Missing data were also listed. Statistical significance was set at *P* < 0.05.

## Results

### Clinical characteristics of the study participants

A total of 30 patients with anti-SRP-associated IMNM were included in the study. Of these, 6 patients (two males, four females) with a median age of 39 years were classified in anti-SRP IMNM overlap SS group, and the remaining 24 patients (ten males, fourteen females) with a median age of 46 years were classified into the the anti-SRP IMNM control group.

Clinical characteristics of the study participants are summarized in Table 1. The prevalence of muscle atrophy was less common in patients with anti-SRP IMNM overlap SS than controls (0 vs 50%, *p* = 0.019). There were no significant differences in age (*p* = 0.264), sex (*p* = 0.545), muscle strength (upper and lower limbs), disease duration (*p* = 0.827), or serological findings between the two groups. The presence of interstitial lung disease (ILD) (*p* = 0.23), cardiac complications (*p* = 0.352), and malignancy (*p* = 0.068) showed no significant difference between the two groups. All patients underwent immunotherapy, and patients in both groups showed either marked improvement (1/6, 16.7% vs 5/24, 20.8%) or improvement (5/6, 83.3% vs 19/24, 73.7%). The proportion of relapse was 16.7% (1/6) and 8.3% (2/24) respectively in the anti-SRP IMNM overlap SS and control groups, respectively.

**Table 1:** Comparison between anti-SRP IMNM overlap SS and anti-SRP IMNM patients

Items	Anti-SRP IMNM-SS (*n* = 6)	Anti-SRP IMNM (*n* = 24)	*P*-value
**Demographics**			
Sex(Female)	4(66.7)	14(58.3)	0.545
Age at myositis diagnosis(years)	39.17 ± 16.216	46.5 ± 13.593	0.264
Disease duration (months)	16 ± 27.67	14.03 ± 17.12	0.827
**Clinical manifestation**			
Upper promixal limbs(median,MRC)	4	4	0.25
Upper distal limbs(median,MRC)	4.5	4	0.3
Lower promixal limbs(median,MRC)	3.5	4	0.36
Lower distal limbs(median,MRC)	4	4	0.17
MMT	206 ± 38.5	199 ± 29.4	0.432
DAS	3.5 ± 2.4	4.9 ± 2.1	0.191
MYOCAT	11.2 ± 7	16 ± 8.9	0.21
Dysphagia	0	8(33.3)	0.155
Dyspnea	0	2(8.3)	> 0.9999
Myalgia	2(33.3)	12(50)	0.657
Atrophy	0	14(50)	**0.019**
Rash	1(16.6)	2(8.3)	0.515
**Examination**			
Thyroid dysfunction	3(50)	9(37.5)	0.66
Rheumatoid factor	0	2(8.3)	> 0.9999
ANA abnormality	5(83.3)	19(79.1)	0.553
Lupus Anticoagulant	0	2(8.3)	> 0.9999
Malignancy	0	10(41.6)	0.068
Statin exposure	0	1(4.2)	> 0.9999
Smoking	0	2(8.3)	> 0.9999
Drinking	0	2(8.3)	> 0.9999
Diabete	0	2(8.3)	> 0.9999
Hypertension	1(16.6)	1(4.2)	0.366
CK(U/l)	6238 ± 3443	3485 ± 4244	0.155
LDH(U/l)	826 ± 524	639 ± 447	0.115
ESR(mm/H)	20.5 ± 6.36	14.79 ± 15.39	0.621
hsCRP(mg/L)	3.38 ± 3.8	16.59 ± 24.19	0.307
NI-PRO-BNP(pg/ml)	18.67 ± 12.662	87.38 ± 82.442	0.177
Alb(g/L)	38.083 ± 5.53	36.84 ± 5.23	0.616
Glb(g/L)	26.72 ± 4.57	30.17 ± 8.05	0.366
WBC(*10^9^/L)	9.45 ± 4.48	10.23 ± 4.77	0.718
Neutrophil(*10^9^/L)	6.9 ± 4.5	7.11 ± 4.3	0.922
Lymphocyte(*10^9^/L)	1.76 ± 0.8	2.5 ± 1.09	0.128
TnI(pg/ml)	17.8 ± 5.14	38.1 ± 45.481	0.113
CK-MB(ng/ml)	562.5 ± 573.464	92.91 ± 83.804	0.453
Myoglobin(ng/ml)	883.6 ± 196.28	401.23 ± 379.846	0.015
GLU(mmol/L)	4.65 ± 0.354	6.8 ± 6.0	0.63
HbA1c(%)	5.35 ± 0.63	6.2 ± 2.2	0.602
ALT (U/l)	134.5 ± 55.2	83.5 ± 67.94	0.104
AST (U/l)	457.83 ± 493.311	110.17 ± 98.0	0.145
Cr(umol/L)	33.6 ± 12.3	42.57 ± 14.67	0.21
C4(g/L)	0.21 ± 0.042	0.19 ± 0.07	0.909
C3(g/L)	0.93 ± 0.172	0.93 ± 0.125	0.972
IgA(g/L)	2.48 ± 0.752	1.99 ± 0.682	0.217
IgG(g/L)	13.83 ± 0.866	12.78 ± 2.79	0.477
IgM(g/L)	1.36 ± 0.646	1.42 ± 0.929	0.906
Interstitial lung disease	2(33.3)	1(4.1)	0.23
Cardiac abnormality	3(50)	5(25.0)	0.352
Ejection Fraction	0.63 ± 0.014	0.68 ± 0.06	0.282
**Treatment**			
Corticosteroids monotherapy	1(16.7)	10(41.7)	/
Corticosteroids plus Immunosuppressant	5(83.3)	14(58.3)	/
**Follow-up**			
Follow-up period, median (range), months	36(6–120)	50(24–90)	0.815
Lost to follow-up	0	1(4.1)	> 0.9999
**Prognosis**			
No improvement	0	0	/
improvement	5(83.3)	19(73.7)	/
Marked improvement	1(16.7)	5(20.8)	/
Relapse	1(16.7)	2(8.3)	/

Data present as n (%) and mean ± SD. *P* values comparing with SS and without SS groups are from x^2^ test, Fisher’s exact test,T test or Mann–Whitney U test. *MRC* Medical Research Council, *CK* creatine kinase, *LDH* lactate dehydrogenase, *AST* Aspartate aminotransferase, *ALT* Alanine transaminase, *Myo* myoglobin, *CK-MB* creatine kinase-MB, *TnI* Troponin I, *ESR* erythrocyte sedimentation rate, *hsCRP* hypersensitive-CRP, *Cr* creatinine, *Alb* albumin, *Glb* globulin, *GLU* glucose, *MMT* Manual Muscle Testing score of 26 muscle groups, *DAS* Disease Activity Score, *MYOACT* Myositis disease activity assessment visual analogue scale

The detailed information of anti-SRP IMNM overlap SS group is shown in Table 2. In two (40%) cases, a diagnosis of ILD was made according to chest computerized tomography findings. Cardiac abnormalities (including myocardial fibrosis, ventricular or atrial dilatation, pericardial effusion, or enlargement of cardiac chambers detected by Echo, or myocardial ischemia detected by cardiac MRI) were observed in three (50%) patients. All six patients received immunotherapy after providing informed consent. Corticosteroid therapy alone was prescribed for one (16.7%) patient, while four (66.7%) patients were treated with corticosteroids and immunosuppression tacrolimus, and one (16.7%) patient initially received corticosteroids with tacrolimus at first, which was changed to rituximab therapy after relapsed. During the follow-up period, one (16.7%) patient relapsed, but the initiation of an intensive treatment was efficient.

**Table 2:** Characteristics of the six anti-SRP IMNM overlap SS patients

	Patient1	Patient2	Patient3	Patient4	Patient5	Patient6
**Demographics**						
Sex (Male/Female)	M	F	F	F	M	F
Age at diagosis of SS (years)	18	26	35	53	42	61
Disease duration (months)	12	2	4	3	72	3
**Diagosis criteria for SS**						
Dry eye	-	-	-	-	-	+
Dry mouth	-	-	-	+	-	-
Shirmer's test	-	NA	NA	NA	-	+
Decreased salivary flow	-	-	-	-	+	+
Parotid swelling	-	-	-	-	-	-
Pathologic salivary gland biopsy ( focal lymphocytic sialad	+	+	+	+	+	+
FS	1	2	1	1	2	2
Anti-SSA	+	+	+	+	+	+
Anti-SSB	+	-	-	-	+	-
Anti-SSA/Ro-52	-	+	-	+	+	+
**Clinical manifestation**						
Upper promixal(MRC ≤ 3)	-	-	-	-	-	+
Upper distal(MRC ≤ 3)	-	-	-	-	-	-
Lower promixal(MRC ≤ 3)	-	-	+	+	-	+
Lower distal(MRC ≤ 3)	-	-	-	-	-	+
MMT	230	184	240	206	235	140
DAS	2	6	2	3	1	7
MYOCAT	16	15	5	5	5	21
Atrophy	-	-	-	-	-	-
Dyspnea	-	-	-	-	-	-
Dysphagia	-	-	-	-	-	-
Myalgia	+	-	-	+	-	+
Thyroid dysfunction	+	-	-	+	-	+
Lupus Anticoagulant	-	-	-	-	-	-
Cutaneous	-	-	-	-	-	+
Statin exposure	-	-	-	-	-	-
Smoking	-	-	-	-	-	-
Drinking	-	-	-	-	-	-
Diabete	-	-	-	-	-	-
Hypertension	-	-	-	-	+	-
Renal	-	-	-	-	-	-
B cell lymphoma	-	-	-	-	-	-
Central nervous system	-	-	-	-	-	-
Peripheral nervous system	-	-	-	-	-	-
Arthritis	-	-	-	-	-	-
Pulmonary	-	+	-	+	-	-
Cardiac abnormality	+	-	-	+	+	-
Vascular	-	-	-	-	-	-
Haematological	-	-	-	-	-	-
Raynaud's phenomena	-	-	-	-	-	-
**Examination**						
ANCA	-	-	-	-	-	-
ANA Titer	+	+	+	+	-	+
ElevatedCK level (U/l)	8419	7853	9516	321	7319	4000
Elevated LDH level (U/l)	1621	410	377	454	1306	793
AST (U/l)	130	148	267	25	136	121
ALT (U/l)	127	144	172	25	368	218
Myoglobin(ng/ml)	729.6	672.6	862.9	> 1200	-	894
CK-MB(ng/ml)	157	64.3	968	115.4	-	-
TnI(pg/ml)	12	19	-	-	22	-
NI-PRO-BNP(pg/ml)	-	-	-	290	-	-
ESR(mm/H)	-	-	-	16	25	-
hsCRP (mg/L)	NA	0.3	0.2	NA	5	8
Alb(g/L)	44.2	33.7	33.7	44.7	32.5	39.7
Glb(g/L)	24.7	34.2	NA	27.9	23	23.8
IgA(g/L)	3.5	2.3	NA	1.69	NA	2.44
IgG(g/L)	15	13	NA	13.4	NA	13.9
IgM(g/L)	1.5	0.89	NA	2.22	NA	0.84
C3(g/L)	1.1	0.69	NA	0.98	NA	0.93
C4(g/L)	0.25	0.16	NA	0.18	NA	0.23
**Pathological examination**						
Labial glands biopsy ( focal lymphocytic sialadenitis with a	+	+	+	+	+	+
**Treatment outcome**						
Immunomodulatory treatment	RTX	TAC,PNL	TAC,PNL	MP	TAC,PNL	TAC,PNL
Follow-up(months)	72	8	6	36	120	36
Dry eye	-	-	-	-	-	-
Dry mouth	-	-	-	-	-	+
No improvement	-	-	-	-	-	-
improvement	+	-	+	+	+	+
Marked improvement	-	+	-	-	-	-
Clinical relapse	+	-	-	-	-	-

*SS* Sjogren’s syndrome, *RTX* rituximab, *PNL* prednisolone, *TAC* Tacrolimus, *MP* Methylprednisolone, *NA* non-available, *FS* Focus Score, *MMT* Manual Muscle Testing score of 26 muscle groups, *DAS* Disease Activity Score; *MYOACT* Myositis disease activity assessment visual analogue scale

### Histological features

**Fig. 1:**
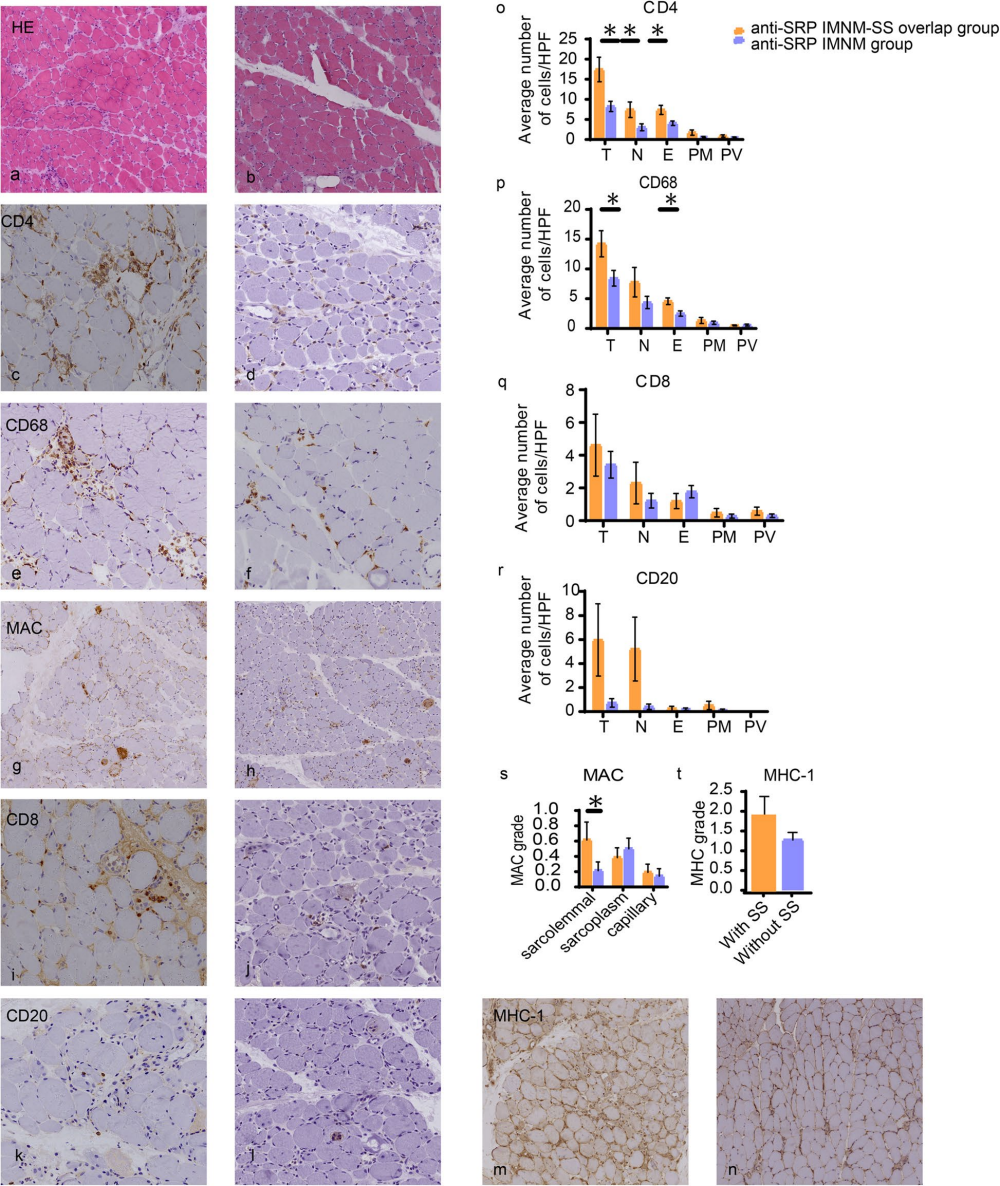
Characteristics of pathological from anti-SRP IMNM overlap SS (**a**, **c**, **e**, **g**, **i**, **k**, **m**) and anti-SRP IMNM patients (**b**, **d**, **f**, **h**, **j**, **l**, **n**). Inflammatory cell infiltrates to the endomysium (E), necrotic(N), perimysial (PM), perivascular (PV) areas. **a**, **b** Representative images of H&E stainings revealed remarkable myofibers necrosis and regenerative in both groups. **c**, **d**, **e**, **f** Representative images of CD4 + and CD68 + of both groups. **o**, **p** The numbers of anti-SRP IMNM overlap SS patients were significantly increased in the presence of CD4 + cells in both necrotic(*p* = 0.023) and endomysial areas (*p* = 0.013), as well as in the presence of CD68 + cells (*p* = 0.016) infiltrated the endomysial area. **g**, **h**, **s** Representative images of sarcolemmal MAC deposition on both groups. Deposition was more commonly seen in anti-SRP IMNM overlap SS patients (*p* = 0.013). **i**-**n** Representative images of CD8 + , CD20 + cells and MHC class I upregulation. **q**, **r**, **t** No significant differences were found between two groups. **a**-**b** scale bar is 250um, (c-n) scale bar is 100um. Data are given as mean with SE. * *P* < 0.05. HPF, high-power fields

All patients underwent muscle biopsy. H&E staining revealed myofibers undergoing degeneration, necrosis, and regeneration as the predominant abnormal features (Fig. 1 a, b). A summary of observed pathological findings is provided in Table 3. Overall, we found several statistical differences in the presence of CD4 + and CD68 + cells (Fig. 1 c-f) between the two groups. CD4 + cells were present in 100% of the anti-SRP IMNM overlap SS group, compared to 54.2% of the anti-SRP IMNM group (*p* = 0.013) (Fig. 1 o). CD68 + cells were found in 100% of the anti-SRP IMNM overlap SS group compared to 50% of the anti-SRP IMNM group (*p* = 0.046) (Fig. 1 p). The significant differences were caused mostly by differences in the presence of CD4 + cells in the necrotic(*p* = 0.023) and endomysial muscle fibers (*p* = 0.013) (Fig. 1 c, d, o), as well as in the presence of CD68 + cells (*p* = 0.016) in the endomysial muscle fibers (Fig. 1 e, f, p). Deposition of MAC on sarcolemma was significantly different between the two groups (*p* = 0.013) (Fig. 1 g, h, s). The presence of CD8 + , CD20 + cells, and the degree of MHC-I upregulation in myofibers showed no significant differences between the two groups (Fig. 1 i, j, k, l, m, n, q, r, t).

**Table 3:** Pathological manifestations of patients in this study

		Anti-SRP IMNM-SS (*n* = 6)	Anti-SRP IMNM (*n* = 24)	*P* value
CD4 (cell count > 5)	Total	6(100)	13(54.2)	0.013
	Necrosis	4(66.7)	4(16.7)	0.023
	Endomysial	5(83.3)	7(29.2)	0.013
	Perimysial	0	0	0.108
	Perivascular	0	0	0.219
CD8 (cell count > 5)	Total	2(33.3)	6(25)	0.614
	Necrosis	3(50)	1(4.2)	0.355
	Endomysial	0	1(4.2)	0.573
	Perimysial	0	0	0.421
	Perivascular	0	0	0.268
CD20 (cell count > 5)	Total	3(50)	1(4.2)	0.176
	Necrosis	2(33.3)	0	0.123
	Endomysial	0	0	0.7
	Perimysial	0	0	0.387
	Perivascular	0	0	> 0.9999
CD68 (cell count > 5)	Total	6(100)	12(50)	0.046
	Necrosis	4(66.7)	8(33.3)	0.176
	Endomysial	2(33.3)	2(8.3)	0.016
	Perimysial	0	0	0.295
	Perivascular	0	0	0.614
MHC(Grading Scales)	Sarcolemmal/Sarcoplasmic staining	5(83.3)	18(75)	0.387
MAC(Grading Scales)	Sarcolemmal	5(83.3)	4(16.7)	0.013
	Sarcoplasm	3(50)	9(37.5)	> 0.9999
	Capillary	3(50)	2(8.3)	0.176

Data presented as n (%). *P* values comparing two groups are from Mann–Whitney U test

**Fig. 2:**
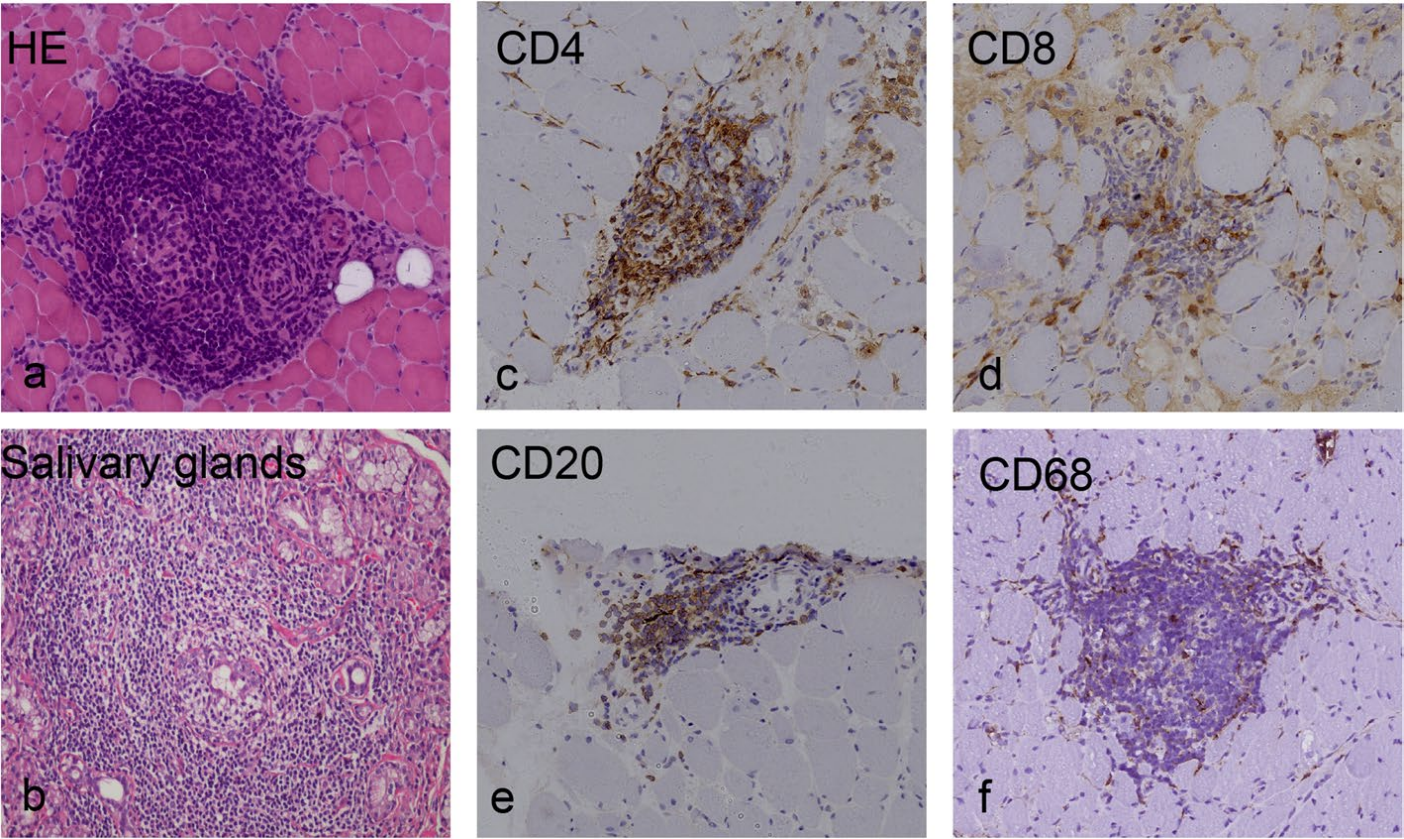
Representative images of one anti-SRP IMNM overlap SS patients showed that inflammatory infiltration gathered into germinal center-like structures. H&E staining and immunostaining: nodular infiltrate with a follicular aspect which was similar to salivary gland biopsy (**b**), compound of T cells (CD4 + and CD8 +) (**c**, **d**) and B cells (CD20 +) (**e**). Scale bar is 100um

**Fig. 3:**
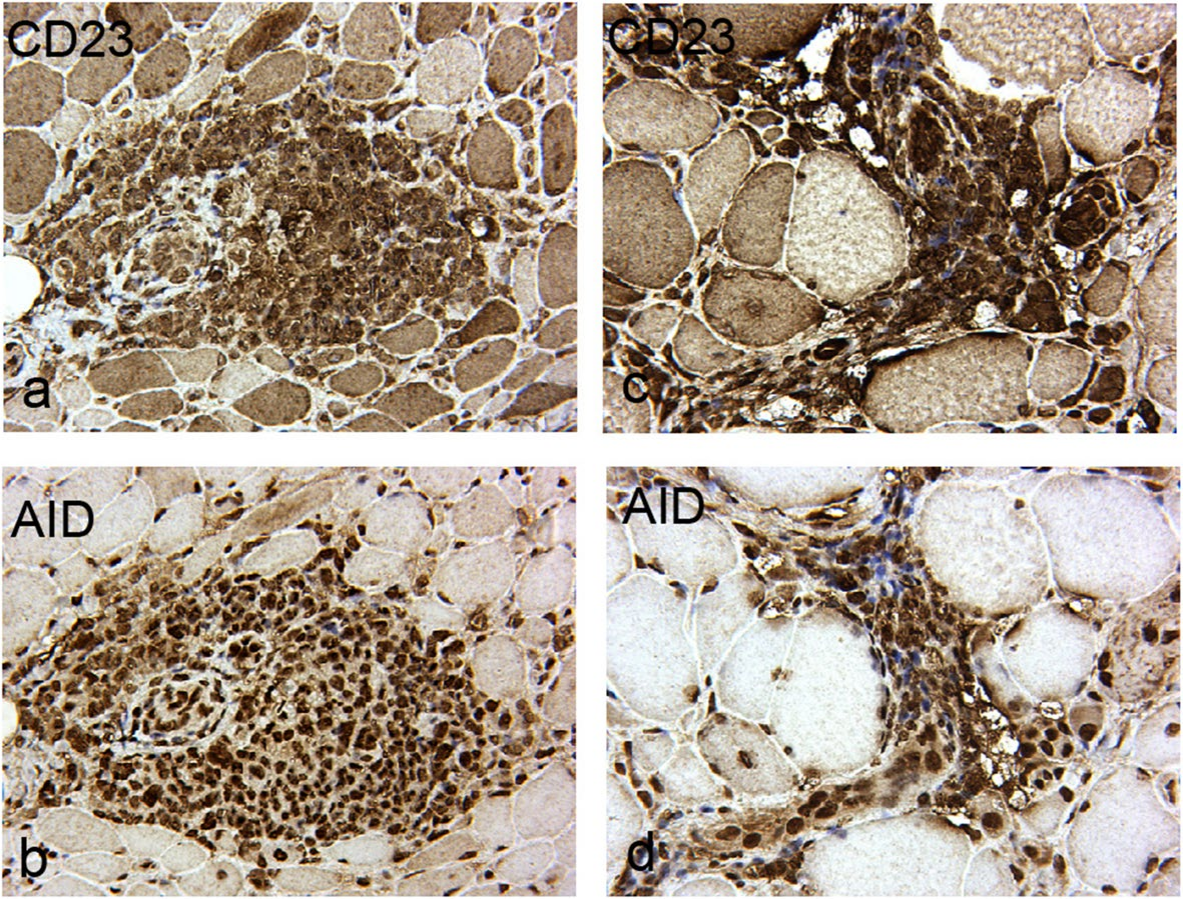
Immunohistochemical images of CD23 + B cells and AID from two anti-SRP IMNM overlap SS patients shown germinal center-like structures. Both showed that abundant lymphoid infiltrates with CD23 + B cells and AID expressed in germinal center-like structures. Images of **a** and **b** were from one patient, **c** and d were from the other patient. Scale bar is 50um

Among patients with anti-SRP IMNM overlap SS, two patients presented with germinal center-like structures of the lymphocytic foci, comprising lymphocytes, AID and macrophages infiltrations grouped into masses (Fig. 2 a, c-f; Fig. 3 a-d). However, this phenomenon was not found in the remaining four patients.

## Discussion

This is the first study to elucidate the clinical manifestations, myopathological characteristics, and prognosis of anti-SRP IMNM-SS overlap patients in China. Herein, we compared the clinical characteristics and myopathological findings of anti-SRP IMNM-SS overlap compared to control anti-SRP IMNM patients. Our data revealed that anti-SRP IMNM-SS overlap patients may present milder clinical symptoms and with a lower prevalence of muscle atrophy compared with anti-SRP IMNM patients. However, CD4 + and CD68 + cells inflammatory infiltrations and MAC deposition are more commonly observed in anti-SRP IMNM-SS overlap patients.

Anti-SRP-associated IMNM is one of the most disabling auto-immune myopathies, characterized by an initially severe muscle weakness as well as often poor muscle recovery, even with treatment [7]. The prevalence of anti-SRP IMNM overlap SS patients in the present study is 0.51% of the total IMNM patients which had not been reported so far. Due to the rarity of this condition, little is known about the clinical and histopathological features of anti-SRP IMNM overlap SS patients. As known as for now, IMNM exhibits a strong type 1 helper T cell (Th1)/classically activated macrophage M1 response, with detection of elevated IFN-γ, IL-12, tumor necrosis factor (TNF)-α and STAT1 levels in the muscle tissue, which may serve as biomarkers in diagnosis [14]. Meanwhile the Th1/Th2 cytokine balance play a central role in the regulation of immunity and dysregulation of SS. It is thought to be a Th1 dominated disease primarily because IFN-γ, IL-12, IL-18, TNF-α, IL-1b, IL-6 and B-cell activating factor (BAFF) are consistently found to be highly expressed in SS patients [15] Otherwise Th2-related cytokines had been also found elevating at the mRNA and (or) protein level in the majority of labial salivary glands (LSG) of patients with SS, such as IL-13 and IL-4 [16, 17]. Muscle atrophy was less frequently observed in anti-SRP IMNM overlap SS patients than anti-SRP IMNM patients in the present study**.** Previous studies have reported that in such patients, muscle regeneration was impaired due to a defect of myoblast fusion associated with a decreased production of cytokines IL-4 and IL-13 in anti-SRP-associated IMNM patients [18]. While cytokines IL-4 and IL-13 were expressed at high frequency in SS patients [16, 17], we suspected that high levels of cytokines IL-4 and IL-13 in anti-SRP IMNM overlap SS patients rescued the down-regulated cytokines levels. This maybe indicating that they represented milder clinical symptom.

In addition to skeletal muscle weakness, the most concerning factor of the extra-muscular manifestations is the risk of cardiac involvement, as the inflammation observed in skeletal muscle can also occur in heart muscle [19]. In some cases, cardiac involvement is severe [7], and may be responsible for sudden death in some patients [20]. Myocarditis was frequently observed in patients with anti-SRP-associated IMNM, occurring in 2–40% of these patients [1]. The prevalence of cardiac abnormalities of anti-SRP IMNM patients (25%) in the present study is consistent with the previous research. Progressive myocardial fibrosis was the most frequently observed abnormality in SS patients [21], and may contribute to a higher incidence of cardiac abnormalities in anti-SRP IMNM-SS overlap patients (50%). ILD is a crucial complication of the IIM [22], which is found in 23–38% anti-SRP seropositivity patients [1]. Pulmonary involvement is very common in SS patients, reported to be found in up to 90% of cases [21]. Higher prevalence of ILD was also observed in anti-SRP IMNM-SS overlap patients (33.3%) than anti-SRP IMNM patients (4.1%). No significant differences in cardiac and lung involvements were found between the two groups.

Thus far, no randomized, blinded, controlled trials investigating patients with IMNM, have been published yet, and most studies investigating medication depended on empirical medication. Initial therapeutic approaches generally comprise corticosteroids; however, some patients further require second-line agents, such as methotrexate and rituximab. Therapeutic strategies for pSS were relief of symptoms as the most important goal [23]. The biologics treatments were used in SS, such as abatacept, rituximab and belimumab [24], and effectively treat extraglandular manifestations [25]. The therapeutic strategies for anti-SRP IMNM-SS overlap were rare reported. In one study steroids, steroids plus MTX were shown to be efficient in achieving remission of myositis associated with primary Sjogren’s syndrome [26]. In our study, all the six patients received immunotherapy, of whom four (66.7%) patients were treated by corticosteroids and tacrolimus. Tacrolimus is a calcineurin inhibitor, which plays a role in cellular immunity, and is effective in preventing relapses in some antibody-mediated autoimmune neurologic diseases [27] including myasthenia gravis [28], NMOSD [29], IMNM [30], rheumatology [31]. Tacrolimus blocks the production of IL-2, and in turn suppresses the activation of T cells, and thereby the production of antibodies by B cells, which requires cross-talk with activated T cells [27]. In our center, tacrolimus has been proved to be effective in IMNM. It is relatively safe when tacrolimus is administered depending on the blood concentration of the agent [30]. In the present study, one (16.7%) patient initially received corticosteroids and immunosuppressive tacrolimus, but was changed to rituximab therapy when he relapsed.

The lower prevalence of muscle atrophy of anti-SRP IMNM overlap SS group prompted us to determine whether the histopathological features of patients with anti-SRP IMNM overlap SS were distinct from those with anti-SRP IMNM. Although previous studies observed inflammation in IIM associated SS patients, the composition of inflammatory cells was not analyzed. Thus, it is the first study to quantitative analyze immunohistochemical stainings of muscle specimens from anti-SRP IMNM overlap SS using a series of cell surface markers to define inflammatory cell subtypes and their distribution. Consistent with previous reports, both groups of patients showed remarkable myofiber necrosis and regenerative, fewer lymphocytic infiltration (mainly CD68 + macrophages), up-regulation of MHC class I molecules expressed in a sporadic manner on sarcolemma surface, and C5b9 sparse deposition on the sarcolemma of myofibers and endomysial capillaries [32]. We further observed remarkable inflammations in both groups, however, the number of CD4 + T and CD68 + cells were much higher in anti-SRP IMNM-SS overlap group than in the anti-SRP IMNM group, especially in necrotic and/or endomysial areas. Previous studies reported remarkable infiltration of T lymphocytes in anti-SRP IMNM, but abundant accumulation was not found [33]. SS is a chronic systemic autoimmune disease characterized by the infiltration of T and B lymphocytes into exocrine glands [34, 35], and CD4 + T cells are mainly observed in the salivary glands and peripheral blood of primary SS (pSS) patients. These cells play a crucial role in the induction and/or maintenance of pSS, such as providing a stimulus for B lymphocytes, and promoting the destruction of the gland through cytotoxicity [36]. Previous literatures had demonstrated that pathological characteristics of SRP-IMNM showed scattered or focal CD68 + macrophage infiltration, scattered CD4 + and CD8 + T lymphocyte, and a few CD20 + B lymphocytes, scattered CD56 + myofiber regeneration as well [37]. We hypothesized that abundant CD4 + T cells in the muscle specimens of anti-SRP IMNM associated with SS might be involved the immunological mechanism, but further mechanistic studies will be required to discuss.

Furthermore, the observation of sarcolemmal MAC deposition in anti-SRP IMNM with SS patients may suggest that humoral immunity may play a major effector role in the pathophysiology of these patients. Measurement of sarcolemmal MAC deposition may be useful in early clinical monitoring, as it is likely to be an initiating or early factor in the process of myonecrosis [38].

Notably, in the present study, two anti-SRP IMNM overlap SS patients who did not receive immunotherapies prior to muscle biopsy had germinal center-like structures, histologically characterized as lymphocytic foci. Infiltrating CD4 + T and CD20 + B lymphocytes gather into masses, replicating the appearance of biopsies in accessory salivary glands (Fig. 2 b). Infiltration of two germinal center associated biomarkers, including CD23 + B lymphocytes and AID, grouped into germinal center-like structures as well (Fig. 3 a-d). This type of lesion had been reported in several cases [4] in PM [26], juvenile DM [39], and sIBM [40]. Nevertheless, this phenomenon was not found in anti-SRP IMNM patients. In the remaining four anti-SRP IMNM overlap SS patients who were administered corticosteroid therapy before muscle biopsy, we speculated that immunotherapy may have inhibited the massive inflammatory response.

## Conclusion

Overall, in this study, we found that anti-SRP IMNM overlap SS may present with milder clinical symptoms with a lower prevalence of muscle atrophy. However, it is difficult to distinguish these patients from anti-SRP IMNM by clinical features alone. Furthermore, we showed that immunotherapy is effective in the treatment of anti-SRP IMNM overlap SS patients. Regarding myopathology, inflammatory infiltrations of CD4 + and CD68 + T cells as well as MAC deposition were more commonly observed in anti-SRP IMNM overlap SS patients. We hypothesize that the germinal center-like structures of the lymphocytic foci and their histologic characteristics might be specific to anti-SRP IMNM-SS overlap patients.

### Limitations

In our center, we don’t have anti-HMGCR and seronegative IMNM patients with overlapping SS. As such, a further study is needed to discuss the clinical and myopathological characteristics of anti-HMGCR and seronegative IMNM patients with overlapping SS. Furthermore, this study had a single-center retrospective design, with limited sample size, therefore studies with a larger sample size are required. Furthermore, we did not perform multiple comparison analysis as no correction was performed in the statistical analysis.

## Supplementary Information

**Additional file 1.**

## Data Availability

The data that support the findings of this study are available from the corresponding author, but restrictions apply to the availability of these data, which were used under license for the current study, and so are not publicly available. Data are available from the authors upon reasonable request and with permission of the Tongji Hospital.

## Abbreviations

ACR: American College of Rheumatology
ENMC: European Neuromuscular Centre
MRC: Medical Research Council
ANA: Anti-nuclear antibody
MAA: Myositis-associated antibodies
MSA: Myositis-specific antibody
ASS: Anti-synthetase syndrome
CK: Creatine kinase
LDH: Lactic dehydrogenase
CTD: Connective tissue disease
SS: Sjogren’s syndrome
IIM: Idiopathic inflammatory myopathies
DM: Dermatomyositis
PM: Polymyositis
IBM: Inclusion body myositis
IMNM: Immune-mediated necrotising myopathy
SRP: Signal recognition particle
HMGCR: Anti-3-hydroxy-3-methylglutaryl-coenzyme A reductase
MAC: Membrane attack complex
MHC: Major histocompatibility complex
MTX: Methotrexate
ILD: Interstitial lung disease
