# Supplementary material for: Anti-signal recognition particle positive necrotizing myopathy-sjogren’s syndrome overlap syndrome: a descriptive study on clinical and myopathology features
Source: BMC Musculoskelet Disord. 2023 Mar 23;24:219. doi: 10.1186/s12891-023-06354-5 (PMC10035234; doi:10.1186/s12891-023-06354-5)
Supplement: Supplementary file 1 — Additional file 1. [file 12891_2023_6354_MOESM1_ESM.pdf]

Additional file1 Grading Scales utilised in histopathological evaluation of muscle

Grade	MHC class I	MAC		
		sarcolemmal	sarcoplasm	capillary
0	No sarcolemmal staining	No positive fibres	No sarcolemmal staining	No capillary staining
1	Small numbers of fibers with sarcolemmal staining [10-30%]	Small numbers of positive fibres [0-30%]	Small numbers of fibres with sarcolemmal staining [0-30%]	Mild capillary staining
2	Moderate numbers of fibers with sarcolemmal staining [31-60%]	Moderate numbers of positive fibres [30-50%]	Moderate numbers of fibres with sarcolemmal staining [30-50%]	Moderate capillary staining
3	Fibers with sarcolemmal staining and scant sarcoplasmic staining [61-90%]	Many positive fibres [>50%]	Many fibres with sarcolemmal staining [>50%]	Widespread capillary staining

MHC class I, major histocompatibility complex class I; MAC, membrane attack complex
